# Carbon and Nitrogen Stable Isotopic Discrimination Factors Between Diet and Feces in Wild Giant Pandas

**DOI:** 10.3390/biology15030274

**Published:** 2026-02-03

**Authors:** Guoyan Long, Yue Wu, Lu Huang, Yonggang Nie, Han Han

**Affiliations:** 1 Liziping Giant Panda’s Ecology and Conservation Observation and Research Station of Sichuan Province, Nanchong 637009, China; 2 Key Laboratory of Southwest China Wildlife Resources Conservation (Ministry of Education), China West Normal University, Nanchong 637009, China; 3 College of Giant Panda, China West Normal University, Nanchong 637009, China; 4 Institute of Zoology, Chinese Academy of Sciences, Beijing 100101, China

**Keywords:** giant pandas, carbon and nitrogen stable isotope, discrimination factor, feces

## Abstract

Stable isotope analysis is very useful for studying animal nutritional ecology. Feces are the most accessible and non-invasive samples for short-term dietary reconstruction. The giant panda is a special Carnivora species with a highly specialized diet. However, no relevant research has yet explored the reliability of fecal isotopes in wild giant pandas, and the key parameter—fecal isotopic discrimination factors—remains unreported. Thus, we analyzed carbon and nitrogen isotopes of different bamboo species and parts with associated pandas’ feces collected from their foraging sites. The results showed carbon isotopes of shoots were more positive than those of leaves, and the isotopic composition of their feces can effectively reflect seasonal dietary shifts. The calculated fecal carbon discrimination factor was close to zero (Δ^13^C_diet-feces_ = 0.6 ± 0.8‰), while the nitrogen DFs were significantly positive (Δ^15^N_diet-feces_ = 2.1 ± 1.2‰). The typical metabolic pattern, physiological adaptations and distinctive microbiota of giant pandas contribute to the unique DFs different from those of other herbivores. These findings provide valuable short-term dietary records, key parameters for the application of fecal isotopes to interpret foraging strategies and nutritional status for an endangered species in the wild, expand the application of stable isotope methods in studies to specialized diet animals, and offer a reference for studies utilizing non-invasive materials in other mammals.

## Simple Summary

This study employed carbon and nitrogen stable isotope analysis to determine the diet–feces discrimination factors (Δ) of wild giant pandas exclusively depending on bamboos. The results showed that the carbon discrimination factor between diet and feces (Δ^13^C_diet–feces_) was approximately zero (0.6 ± 0.8‰), while nitrogen was significantly enriched in feces (Δ^15^N_diet–feces_ = 2.1 ± 1.2‰). These distinct values, which differ from those of typical herbivores, reflect the adaptation of the panda’s carnivore-derived digestive system to its highly specialized diet. This study demonstrated that panda fecal carbon isotopes can reliably record dietary shifts and provided crucial background data for applying stable isotope analysis to investigate the foraging ecology and conservation of this unique species.

## 1. Introduction

Stable isotope analysis (SIA), as an efficient and non-destructive technique, has become a crucial and powerful tool for studying nutritional ecology of animals [1]. It has been used to study various species, including elephants *(Loxodonta africana)* [2], wild mountain gorillas (*Gorilla beringei*) [3], and fin whales (*Balaenoptera physalus*) [4]. Traditional methods for reconstructing animal diets, such as direct field observation, fecal analysis, and stomach/intestinal contents analysis, have biases and obtain limited information respectively [5,6]. The stable isotopic information of an organism’s dietary strategy can be documented in its tissues over short or long periods, avoiding time-consuming and costly field observation [7]. Meanwhile, by utilizing noninvasive tissues like hair or nails, carbon and nitrogen stable isotope composition of an organism’s dietary strategy can be documented in their tissues over short or long periods, avoiding time-consuming and costly field observation [8]. This approach characterizes the entire ingested and assimilated diet across both temporal and spatial scales, facilitating investigations into niche partitioning, nutritional stress, individual dietary variability, as well as temporal or seasonal shifts in diet [8,9]. It is highly valuable for tracing the dietary sources of mammals and monitoring dietary changes across seasons and years, thereby indicating adaptive management strategies. Stable carbon and nitrogen isotope ratios can reveal whether an individual is primarily consuming plants, insects or other animals, its associated ecological niche and ecosystem role [3,10]. By comparing isotopic signatures across different tissues and time periods, researchers can construct a comprehensive picture of how mammals adapt to fluctuating food availability and environmental conditions.

Isotopic fractionation originates from the mass-dependent differences in the physical and chemical behavior of isotopes, resulting in a clear discrepancy in isotopic composition between initial reactant substrates and final reaction products [4]. During assimilation, isotopic fractionation causes animals to preferentially excrete the lighter isotopes (^12^C and ^14^N) and retain the heavier ones (^13^C and ^15^N) in their tissues. This retention results in a progressive enrichment of the heavier isotopes by each elevating trophic level. Consequently, the stable isotopic composition of a consumer diverges from that of its food resources [11] and becomes a valuable tool for ecologists to trace the flow of energy and nutrients through food webs. This concept of carbon and nitrogen isotopic enrichment in mammalian tissues also has implications for understanding the diet and feeding habits of specific animals [12,13,14,15,16]. The time scale of SIA in animal tissues depends on tissue synthesis rates. Bulk samples, such as bone, tooth enamel and hair, can provide integrative information of diet over their formation period—typically months to years [17]. However, when conducting research on extant species using tissue samples such as hair, bones, and blood, certain limitations exist. These include the accessibility of samples and the challenges associated with repeated sampling from the same individual. This is particularly difficult for wild animals, especially rare and endangered species. Additionally, signal attenuation occurs in these tissue types due to extended formation times and a lag in the equilibration of body pools [18]. Feces are easier to obtain and the least invasive sample type in the wild. In contrast with fecal DNA analysis, which provides a high-resolution taxonomic list of undigested and partially digested items, the stable isotopic composition of feces can be applied to short-term dietary reconstruction that includes undigested material and complete physiological pathway—from ingestion and digestion to microbial fermentation and host metabolism and excretion [10]. Therefore, their application in dietary investigations of rare or elusive animals is increasingly prevalent [19]. Fecal stable isotopes mainly derived from undigested food, gut microbes, digestive secretions and exfoliated epithelial tissues have been shown to reflect the ingested diet of mammalian animals in both controlled experimental and natural environments [3,20,21,22,23,24]. SIA in feces has already been applied in studies of some animals. For instance, fecal isotopes have been demonstrated to be an effective method for the rapid and non-invasive monitoring of short-term dietary changes in four South African herbivore species [25]. Meanwhile, fecal nitrogen isotope analysis (δ^15^N) serves as a reliable tool for tracing and quantifying dietary composition in mammalian herbivores, offering a robust method for ecological research on this group [26]. Additionally, fecal carbon and nitrogen isotopes have been employed to assess dietary patterns in social primates such as chimpanzees [27].

The giant panda has undergone dietary shifts during its long evolutionary history, changing from an omnivore to a highly specialized diet almost depending on bamboo [28]. As seasonal altitudinal migratory animals, giant pandas shift between bamboo shoots and leaves related to the concentrations and balances of calcium, phosphorus and nitrogen [29,30]. Although feces can only reflect an animal’s diet within a very short period, they still represent the most promising material for precisely characterizing dietary composition. However, it is well established in the academic community that stable isotope discrimination factors (DFs: difference in isotopic ratios between consumers’ diet and their tissues) constitute the primary limitation for achieving accurate and reliable dietary reconstruction via stable isotope analysis [12,13,14]. A previous study has measured the carbon and nitrogen DFs between giant panda enamel, hair keratin, bone collagen and their diet [31]. However, there is no relevant data regarding the DFs for giant pandas from diet to feces. Therefore, we ascertained the carbon and nitrogen isotopic compositions of different bamboo parts and fecal samples from wild giant pandas (the Qinling Mountains) to verify the reliability of fecal isotopes in reflecting the giant panda’s diet under natural conditions and to calculate the carbon and nitrogen isotopic DFs between diet and feces. The results will provide essential parameters for future fecal stable isotope studies on giant panda, expand the application of stable isotope methods in studies on endangered wild mammals, and offer a reference for studies utilizing non-invasive materials in other species.

## 2. Materials and Methods

### 2.1. Study Site

We conducted this research within Foping Nature Reserve (N 33°32′–33°45′, E 107°40′–107°55′) situated in the Qinling Mountains of Shaanxi Province, China, with elevations ranging from 980 to 2904 m above sea level (a.s.l.). The reserve encompasses 293 km^2^ and was established primarily for the preservation of giant pandas in 1978. The annual mean temperature here is 11.5 °C (min: −3 °C in January, max: 28 °C in July). The annual rainfall is approximately 930 mm and mainly occurs during July and September. Snowfall typically first occurs in November at elevations above 2000 m and then at lower elevations.

The giant panda, as a flagship species of global biodiversity conservation, has long been widely concerned and beloved by the public. It is endemic to China and distributed along the eastern Tibetan Plateau in dense temperate montane forests where the understory is dominated by bamboo. The Qinling Mountains contain a high density of wild giant pandas with a population of over three hundred individuals [31]. Two bamboo species, wood bamboo (*Bashania fargesii*, Bf) and arrow bamboo (*Fargesia qinlingensis*, Fq) are the main diet resources of pandas there. Bf grows at 980–1800 m a.s.l., and Fq grows at 1800–2600 m a.s.l. Giant pandas live at lower elevations from September to June and move to higher ground for the rest of the year. Bf shoots usually start sprouting in May and grow to abundant new leaves in August. By comparison, Fq shoots mostly occur in early June but start developing a few new leaves in the following spring and more in summer [29,32].

### 2.2. Diet and Feces Samples

We used archived scats from a previous study to determine whether stable isotope analysis of giant panda scats might be useful to characterize diet. The fecal samples of giant pandas were opportunistically collected from the wild field between March 2011 and February 2012 with pair-sampled bamboo diet at the foraging sites. Giant pandas in this region consume different parts of bamboo species consistent with their reproductive cycles. In early May, when Bf shoots reached sufficient height for consumption, pandas shifted to feed on them. Then pandas migrated to higher elevations and began to eat Fq shoots in early June as Bf shoots grew tall and became lignified at that time. Subsequently, pandas switched to eating Fq leaves when the shoots of them grew tall. In September, pandas went back to forage Bf leaves until the next May for new shoots. Moreover, pandas preferred the younger leaves during the two leaf periods [32]. A total of 122 fecal samples were collected, along with 83 subsamples of diet items: shoots and leaves of Bf and Fq, respectively. It has been reported that maternal milk can influence isotopic results [33]. Age classification was based on the fecal morphological characteristics. Four age-groups could be identified by measuring the bite-sizes of panda droppings: cubs, juveniles, adults and seniors [34]. Only feces of adults with no obvious desiccation were collected to ensure that isotopic signatures were not affected by contamination. Each sample was placed into a zip-lock plastic bag, and information was recorded including date and location. The scats were frozen until future analyses.

### 2.3. Stable Isotope Analysis

All samples were dried at 60 °C in the oven and ground to powder with a common multifunctional laboratory mill before laboratory analysis. Bamboo (approximately 2 mg) and feces (1–2 mg) were put into tin capsules and combusted on a Flash Elemental Analyzer 1112 (Thermo Fisher Scientific, Waltham, MA, USA). Carbon and nitrogen stable isotope ratios of resulting gases were measured with a MAT253 isotope ratio mass spectrometer (Thermo Fisher Scientific, Waltham, MA, USA) at the Institute of Zoology, Chinese Academy of Sciences, Beijing, China. C and N content (the percentage of carbon and nitrogen in the samples) was determined by the elemental analyzer, with an error of 1% for carbon and 2% for nitrogen. The lab standard used to measure the contents of C and N was Urea (IVA33802174, C% = 20%, N% = 46.6%, Thermo Fisher Scientific, Waltham, MA, USA). IAEA-600 (IAEA, Vienna, Austria, δ^13^C = −27.8‰, δ^15^N = 1.0‰) was used to normalize N_2_ and CO_2_ in steel bottles. All stable isotope ratios of the samples are reported as the conventional delta (δ) values: δ_sample_ = [(R_sample_/R_standard_) − 1] × 1000, where the δ_sample_ is the isotope ratio of the sample relative to the standard, R_sample_ and R_standard_ are the fractions of heavy to light isotopes (^13^C/^12^C and ^15^N/^14^N) in the sample and standard, respectively. δ values for carbon and nitrogen are reported in permil (‰) notation relative to V-PDB (Vienna-Pee Dee Belemnite limestone) and AIR (atmospheric air), respectively. Repeated isotope analysis of international standard was made to measure analytical precision, which was <0.2‰ for both δ^13^C and δ^15^N.

### 2.4. Trophic Discrimination Factors

Fecal discrimination factors (DFs, Δ) were calculated using monthly average measured isotopic composition of feces and the diet for the δ^13^C and δ^15^N values. We use the simple method to work out isotopic discrimination between diet and animal tissue, “big delta” values: Δ_diet–feces_ = δX_feces_ − δX_diet_. This approximate value is accurate when isotopic differences between two materials are less than 10‰ [35]. A literature review was conducted to gather carbon and nitrogen fecal DFs from other studies for comparison. It is noteworthy that, during data compilation, the formulas used to calculate isotopic DFs varied among studies, mainly two conventions: feces–diet or diet–feces. To guarantee internal consistency, all DF values in this study, including the cited data, have been standardized to the definition: Δ_diet–feces_ = δX_feces_ − δX_diet_.

### 2.5. Statistical Analyses

The normality of the data was examined using the Wilk–Shapiro test. The results indicated that all statistical analyses could be based on parametric statistical tests with normal distributions. Multivariate analyses of variance (ANOVA) with Tukey’s honest significant difference (HSD) pairwise tests in SPSS (version 18.0, SPSS, Chicago, IL, USA) were employed to compare the isotopic composition of forage classes among different bamboo species and tissue parts. A parametric analysis of variance was conducted to test for significant monthly variation in the pooled data of δ^13^C, δ^15^N, C% and N% values of diet and feces. Independent sample tests were performed to detect variances in the pandas’ dietary and fecal isotopic results throughout the entire period. All means of the results were recorded along with standard deviations (M ± SD). For all analyses, the significance level was set at *p* ≤ 0.05.

## 3. Results

### 3.1. Stable Isotopes of Panda Diet

We conducted measurements on the carbon and nitrogen stable isotopic composition and atomic concentration of leaves and shoots of Bf and Fq collected monthly, consistent with pandas’ diet (Table S1a). δ^13^C values were consistent with the expectations for plants following the C_3_-photosynthetic pathway (combined mean = −25.5 ± 1.6‰, *n* = 83). The mean δ^13^C value for each food item was presented as follows: Bf leaves, −30.4 ± 0.9‰ (*n* = 53); Bf shoots, −24.6 ± 0.7‰ (*n* = 22); Fq leaves, −30.5‰ (*n* = 2); and Fq shoots, −26.1 ± 0.3 ‰ (*n* = 6). The carbon isotope ratio average for all bamboo diet items was −29.1 ± 2.4 ‰ (*n* = 83). The mean δ^15^N values are: Bf leaves, −0.6 ± 1.2‰; Bf shoots, −1.1 ± 1.1‰; Fq leaves, −2.4‰; and Fq shoots, 0.8 ± 0.4‰. The nitrogen isotope ratio average for all items was −0.7 ± 0.8‰. Through multiple-comparisons analyses, significant differences were detected in the δ^13^C (F = 81.942, *p* = 0.000) and δ^15^N (F = 4.379, *p* = 0.000) values among different months (Table S3a). There was no marked difference in the stable isotopes of either δ^13^C or δ^15^N between the two bamboo species (Table S3b). However, the δ^13^C values reached a peak during May, June and July, showing that the mean carbon isotopes of bamboo shoots (−24.9 ± 0.9‰, *n* = 28) were more positive than those of leaves (−30.4 ± 0.9‰, *n* = 55, independent *t*-test, F = 0.594, df = 83, *p* = 0.000); while the mean and variance of their δ^15^N values were practically equal (shoots: −0.7 ± 1.3‰, leaves: −0.7 ± 1.3‰) (Table S3c). We also identified statistically significant differences in the carbon percentage (C%) and nitrogen percentage (N%) of bamboos in certain months (Table S3a). Bf shoots from May had the highest nitrogen content (5.4 ± 0.8%, *n* = 16) and the lowest carbon content (41.9 ± 1.6%) throughout the year. Thus, they were richer in nitrogen compared to Bf leaves, Fq shoots and leaves. Generally, bamboo shoots had much larger C/N ratios than leaves. The difference in the C/N ratio between Bf and Fq leaves was not significant (F_(53,2)_ = 0.246, *p* = 0.423), whereas Bf shoots were significantly different from Fq shoots (F_(22,6)_ = 17.328, *p* = 0.000, Table S3b and Figure S1). The mean diet C/N ratio was 15.5 ± 4.7.

### 3.2. Stable Isotopes of Giant Panda Feces

**Figure 1:**
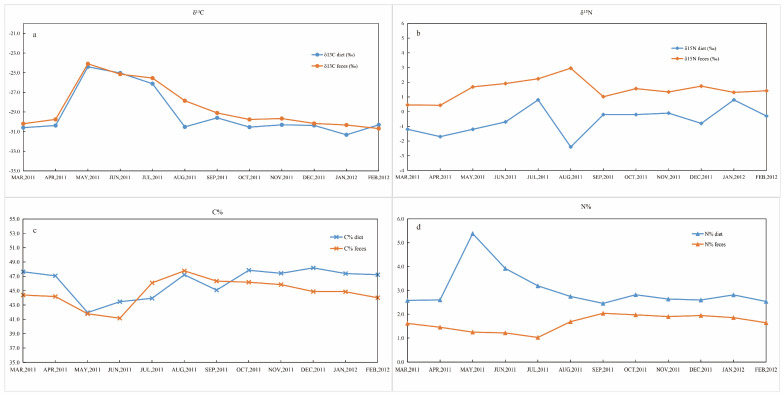
Monthly variations in the isotopic composition of giant pandas’ diet and feces in Foping National Nature Reserve from March 2011 to February 2012 (**a**–**d**) correspond to δ^13^C, δ^15^N, C%, and N%, respectively.

The monthly mean and variabilities of δ^13^C, δ^15^N, C%, and N% for all fecal samples are presented in Table S1b. The panda feces had a wide range of δ^13^C values, from −31.7 to −22.5‰. The mean δ^13^C value for these pandas’ feces was −28.5‰, and the standard deviation was 2.3‰. The results of ANOVA multiple comparison using Tukey HSD exhibited that there were statistically significant differences among the average monthly δ^13^C values of feces (F = 97.446, *p* = 0.000, Table S4a). There was a uniformly elevated δ^13^C value in feces collected during May, June and July (−24.1 ± 0.8‰, −25.2 ± 0.5‰, −25.5± 0.6‰, respectively), which were higher than those of feces collected in other months (F = 97.446, *p* = 0.000, Table S4a). The occurrence of these peaks corresponded to the increased δ^13^C values of bamboo shoots (Figure 1).

The range of δ^15^N values for feces across the entire period was −3.7 to 6.3‰. The mean fecal δ^15^N value was 1.5‰, and the standard deviation was 0.7‰. Mean (± SD) δ^15^N values of Bf leaves and shoot period feces were 1.1 ± 1.4‰ and 1.7 ± 1.2‰ respectively, and corresponding Fq periods δ^15^N values were 2.2 ± 3.1‰ for leaves and 3.0 ± 1.0‰ for shoots. Feces collected in different months did not reach statistical significance in terms of nitrogen isotope ratios (F = 1.867, *p* = 0.051, Table S4b). Temporal variation in feces δ^15^N values was basically correlated with food, except in August (Figure 1), possibly due to the small sample size (*n* = 2) in this month.

The mean C% in feces consuming Bf leaves and shoots was 41.6 ± 2.0% (*n* = 84) and 45.0 ± 1.8% (*n* = 28), respectively; the average C% in Fq shoots was 46.1 ± 1.2% (*n* = 8) and in Fq leaves was 47.8 (*n* = 2, Table S1b). Statistical results indicated that C% of Bf shoots feces was the lowest among the other three food items. The difference in N% between Bf (1.8 ± 0.4%) and Fq leaves (1.7%) feces was significant (post hoc Tukey’s honestly significant difference HSD, *p* = 0.0.005); and the mean N% of Bf (1.2 ± 0.2%) and Fq shoot feces (1.0 ± 0.1%) was also statistical different (Tukey HSD, *p* = 0.000, Table S4c). Nevertheless, there was an obvious difference between fecal shoots and leaves in N% (Table S4c). The average C/N ratio was 30.2 ± 12.1.

### 3.3. Trophic Discrimination Factors

We compared the δ^13^C and δ^15^N values between diet and feces to confirm whether there was a significant difference. Prior to hypothesis testing, a Levene’s Test was performed for equality of variances to determine if a *t*-test for equal or unequal variance should be employed based on the results. The test indicated that the variance was equal for comparisons between feces and bamboos in the case of δ^13^C (F =1.924, *p* = 0.167), while the variance of δ^15^N was significantly different (F = 4.443, *p* = 0.036). Consequently, the subsequent analysis revealed the δ^13^C values between diet and feces were not significantly different (t = −0.637, df = 208, *p* = 0.525), but the δ^15^N values exhibited a marked difference (t = −10.381, df = 203.112, *p* = 0.000, Table S5).

According to previous studies [29,30,31,32], during the sampling period, the foraging pattern of giant pandas was as follows: 8 months for Bf leaves, 2 months for Bf shoots, 1 month for Fq shoots and 1 month for Fq leaves, accounting for 66.7%, 16.7%, 8.3% and 8.3% of the giant panda diet, respectively. Then the weighted δ^13^C and δ^15^N values of pandas’ diet were −28.3 ± 0.7‰ and −0.7 ± 1.0 ‰, respectively (Table S2). The mean δ^13^C and δ^15^N values of all fecal samples were −28.4 ± 2.6‰ and 1.3 ± 1.6‰ (*n* = 122). Hence, the simple calculation of isotopic discrimination from diet to feces was −0.1‰ for δ^13^C and 2.0‰ for δ^15^N. To investigate the fluctuation, discrimination factors were calculated monthly by using average isotopic values of individual feces, subtracting the average value of food samples. The average of 12 months for fecal discrimination factors was ∆^13^C_diet–feces_, 0.6 ±0.8‰, and ∆^15^N_diet–feces_, 2.1 ± 1.2‰ (Table 1 and Figure S2), and only the feces δ^15^N values had monthly increments.

## 4. Discussion

### 4.1. Stable Isotopes in Bamboos

The isotopic composition of tissues is largely dependent on their food sources, such that isotopic variations in bamboos directly influence the fecal isotopic composition of giant pandas. The δ^13^C values of the two bamboo species align with the range characteristic of C_3_ photosynthetic plants and are comparable to the values previously reported from relevant data in the Foping National Nature Reserve [31]. Intraspecific variation in δ^13^C values is observed in plants, and this variation has been documented in animal fecal records [11]. The δ^13^C values of bamboo shoots from both species were higher than those of the leaves, which may be attributed to the “canopy effect”—the closer plants to the forest floor are, the more prominent ^13^C enriched in their tissues tends to be [36]. The mean δ^15^N values of bamboos were similar to those previously researched in the same region but lower than the δ^15^N values of common plants from other sites with dense forest canopy [37]. Within monsoon and tropical rainforests, the δ^15^N values of these plants frequently fall within the range of 3–5‰ [38]. The carbon-to-nitrogen (C/N) ratio in plants serves as an indicator of their internal nutritional quality. The difference in C/N ratios between Bf and Fq shoots indicates why giant pandas frequently switch between different bamboo species and diverse tissues. This dietary alternation enables them to fulfill their nutritional and energy requirements through seasonal foraging strategies, thereby achieving a nutritional equilibrium [29,30,32].

### 4.2. The Giant Panda Feces

The findings suggest that the monthly mean fecal δ^13^C values efficiently reflect the foraging strategy of giant pandas (Figure 1a), therefore demonstrating seasonal fluctuations in the diet of pandas. In comparison to leaves, bamboo shoots display distinct differences in carbon isotopes; consequently, the peak in fecal δ^13^C values can be consistent with the bamboo shoots consumption period. The strong correspondence between the weighted dietary carbon and nitrogen isotope values and those of the feces further validates the dietary sources. Compared to body tissue samples such as teeth, hair, and bones reported in other studies, fecal δ^13^C values were the lowest. Feces, as the end-product of metabolism, have their composition and isotopic signature affected by a series of biological and chemical processes. These processes have greater complexity and variability than those involved in the direct formation of body tissues [31,39]. The integrated analysis of field behavioral observations and fecal samples confirmed that the stable carbon isotope ratios in wild giant panda feces documented their dietary shifts throughout the year, reflecting distinct seasonal variations in the diet. Therefore, fecal δ^13^C data can faithfully record dietary signals and function as a reliable tracer. Measuring the daily variations in fecal δ^13^C allows for precise identification of the timing of major dietary shifts. Although a certain degree of signal attenuation occurs, fecal isotopes compared to observational data retain temporal information on dietary changes at a higher resolution and are more practical and accessible [40].

While the carbon isotopic composition in feces directly reflects dietary intake, the abundance of ^15^N is influenced not only by the isotopic signature of the ingested food but also by additional factors such as the nitrogen content percentage (N%) [41]. Many omnivores and herbivores optimize their food intake by consuming a variety of food types: elephants attain dietary diversity by switching between grazing on grasses and browsing on leaves or shrubs [22]. It is not only a seasonal adaptation to the phenologically driven fluctuations but also allows for an increased reliance on one resource when the other fails to offer sufficient dietary variety [2,22]. Giant pandas similarly alternate between bamboo shoots and leaves, resulting in substantial fluctuations in the nitrogen content of their diet. Although the nitrogen percentage is an indicator of crude protein, fecal nitrogen can also represent undigested dietary nitrogen, endogenous nitrogen, microbial nitrogen, as well as nitrogen bounded to fiber [5,6].

### 4.3. Giant Panda Diet–Feces Discrimination Factors

**Figure 2:**
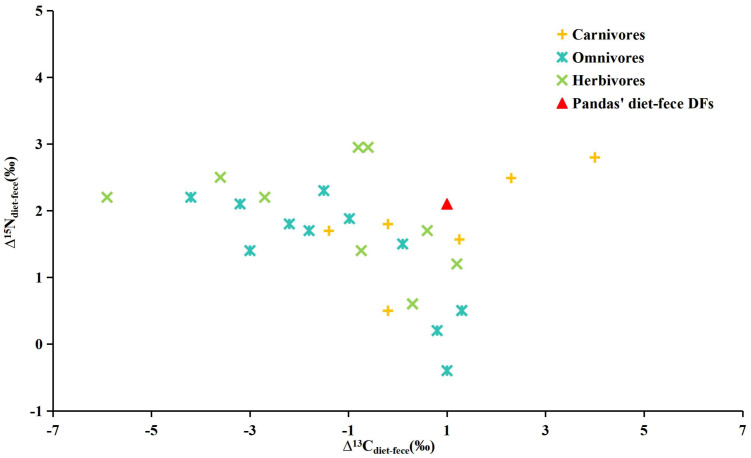
Comparison of diet–feces carbon and nitrogen isotope discrimination factors (Δ^13^C_diet–feces_ and Δ^15^N_diet–feces_) between giant pandas and other mammalian groups (Carnivores, Omnivores, Herbivores). Data sources: Carnivores data from [42,47,48,49]; Omnivores data from [21,23,27,42,50,51,52,53]; Herbivores data from [3,20,21,26,54,55]; Giant pandas from this study.

In herbivores, the Δ^13^C_diet–feces_ are typically negative [42]. However, the δ^13^C offset between diet and feces calculated for wild giant pandas (0.6 ± 0.8‰, Figure 2) is greater than that of other large mammals. For instance, the values for *Capra hircus*, *Gorilla gorilla* and *Equus caballus* were reported as −0.8 ± 0.1‰, 0.3‰ and −0.7 ± 0.2‰ [26,42]. In addition, there are no significant statistically difference among most herbivorous carbon diet–feces discrimination factors (DFs_diet–feces_) [23]. Fecal δ^13^C values can directly indicate the dietary δ^13^C input, as mammals generally show minimal or almost none carbon isotopic discrimination from diet to feces. This is consistent with the results of modern sloth fecal analysis [26,43]. In contrast to herbivores, carnivores consume diets with relatively higher lipid content. If lipids are not sufficiently removed from these dietary samples before analysis, the Δ^13^C_diet–feces_ will be artificially inflated [17,44]. Before the species-specific values are calculated, approximate values of species with similar diets and body sizes are generally cited for analysis. However, the carbon DF_diet–feces_ value of giant pandas shows more comparability with those of carnivores rather than herbivores or omnivores (Figure 2), which is consistent with its taxonomic status. This pattern may be attributed to the fact that, although the giant panda has evolved into an obligate herbivore specialized in bamboo consumption, it still retains a gastrointestinal system of carnivores featuring a relatively long mid-gut and a simple stomach structure. Unlike typical herbivorous mammals that rely on extended gastric fermentation to decompose cellulose, a process that often generates substantial methane, the giant panda adopts a different digestive strategy [45]. Furthermore, the giant panda demonstrates low gut microbial diversity and a phylogenetic composition that is distinguishable from other herbivores, notably lacking the cellulolytic phenotype [46]. This distinctive microbiota is considered a key factor contributing to the species’ low digestive efficiency and the observed carbon isotope enrichment.

Compared to carbon isotopes, nitrogen isotopes exhibit significant isotopic enrichment in feces (Δ^15^N_diet–feces_ = 2.1 ± 1.2‰). The obvious nitrogen discrimination factor may originate from gut tissues, undigested plant fibers, or the activity of gut microbiota [22]. The δ^15^N values in giant panda feces, consistent with other tissues (hair and bone collagen), are substantially enriched relative to the diet, which suggests that the majority of fecal nitrogen is derived from animal-derived sources rather than undigested food. Studies on captive ruminants have found that 60–80% of the nitrogen in their feces is endogenous [19]. The research of digestive tracts of small mammals has revealed that digesta in the stomach, intestine, cecum, and colon are all enriched in ^15^N relative to their diet. This discovery further supports the hypothesis that the enrichment of ^15^N in feces might be attributed to the existence of endogenous proteins [27]. Moreover, unlike carbon, which can be repeatedly mobilized and recycled among all organic compounds within an animal’s body, nitrogen is predominantly partitioned into proteins. When proteins are degraded, they are excreted. Consequently, aside from the initial fractionation that occurs when dietary nutrients are incorporated into tissues, subsequent physiological processes have minimally altered the nitrogen isotope signature [49]. One hypothesis suggests that this species-specific variation (across carnivores, omnivores, and herbivores) may be associated with differences in the animals’ metabolic and digestive traits [4]. The variation in stable isotope ratios within the digestive tract is primarily regulated by two interrelated processes: the decomposition of substrates by bacteria and digestive enzymes and the selective absorption of lighter stable isotopes. These processes are likely adjusted by factors such as dietary composition and the structure and function of the digestive tract, thus leading to the observed variations among herbivores, carnivores, and omnivores.

The carbon and nitrogen stable isotope diet–feces DFs of giant pandas in the wild presumably stem from their specialized physiological adaptations as carnivores to a high-fiber, low-protein bamboo diet, including efficient carbon assimilation, enhanced protein metabolism and urea recycling. Although data from wild individuals circumvented the dietary oversimplification associated with captive settings and possessed greater ecological relevance, the inability to precisely control individual diets and account for confounding variables such as age, health status and season may introduce uncertainties into the accurate calculation of discrimination factors. Furthermore, when conducting comparisons with a wide range of mammals with varying body sizes and digestive strategies, the potential biases resulting from these phylogenetic disparities were not quantitatively evaluated.

The diet–feces isotope discrimination factors established here enable the use of fecal isotopes to study giant panda foraging ecology. A limitation exists in that this method is unable to differentiate the consumption of the two bamboo species (*Bashania fargesii* and *Fargesia qinlingensis*), owing to the highly comparable δ^13^C and δ^15^N values of their leaves. However, this approach can effectively distinguish shoots versus leaves periods as shoots growing in the understory have significantly higher δ^13^C values than leaves from the canopy. This natural isotopic difference is much larger than the discrimination introduced by digestion. Therefore, applying the Δ^13^C_diet–feces_ value allows fecal carbon isotopes to trace the seasonal dietary shifts between leaves and shoots, which is crucial for understanding their foraging ecology and habitat use.

In summary, fecal carbon and nitrogen isotopes with diet–feces discrimination factors are robust, non-invasive indicators for tracking changes in shoot versus leaf consumption of giant pandas in the wild, complementing longer-term dietary signals from tissues such as hair or bone.

## 5. Conclusions

This study employed stable isotope analysis to investigate the diet to feces discrimination factors in giant pandas, revealing that: (1) The carbon discrimination factor (Δ^13^C_diet–feces_) was 0.6 ± 0.8‰, while the nitrogen discrimination factor (∆^15^N_diet–feces_) was 2.1 ± 1.2‰. These values were significantly different from zero, indicating a consistent enrichment of ^13^C and ^15^N in feces; (2) The giant panda with an obligate herbivorous diet demonstrated a near-zero Δ^13^C_diet–feces_, reflecting specialized digestive adaptations to process its high-fiber bamboo diet. Concurrently, the significantly positive ∆^15^N_diet–feces_ implied enhanced protein metabolism and highly efficient urea recycling mechanisms, which are crucial adaptations to its low-protein bamboo diet. (3) The study also found that the carbon isotopic composition of giant panda feces directly reflected their diet, whereas the abundance of nitrogen isotopes is influenced not only by the isotopic signature of ingested food but also by the percentage of nitrogen content (N%). These isotopic data improved the understanding of seasonal foraging patterns in giant pandas. (4) The low gut microbial diversity in giant pandas may contribute to their digestive inefficiency and carbon DFs. Furthermore, nitrogen DFs in feces may be related to gut tissues, undigested plant fibers, or gut microbiota. In summary, the stable carbon and nitrogen isotope ratios in giant panda feces provide valuable short-term dietary records, which are crucial for interpreting their foraging strategies and nutritional status. This study mainly characterized the apparent fractionation patterns in the wild; the underlying physiological mechanisms, such as the functional roles of gut microbiota and the host’s nitrogen metabolic efficiency, await further investigation and elucidation.

## Figures and Tables

**Table 1:** The monthly diet–feces discrimination factors of the giant panda.

Month	δ^13^C Feces (‰)	δ^15^N Feces (‰)	∆^13^C_feces-diet_	∆^15^N_diet-feces (‰)_
MAR, 2011	−30.2	0.5	0.4	1.7
APR, 2011	−29.8	0.3	0.6	2.0
MAY, 2011	−24.1	1.7	0.3	2.9
JUN, 2011	−25.2	1.9	−0.1	2.6
JUL, 2011	−25.5	2.2	0.6	1.5
AUG, 2011	−27.9	3.0	2.7	5.3
SEP, 2011	−29.1	1.0	0.5	0.6
OCT, 2011	−29.8	1.6	0.8	1.8
NOV, 2011	−29.7	1.3	0.6	1.4
DEC, 2011	−30.2	1.7	0.2	2.5
JAN, 2012	−30.3	1.3	1.0	0.5
FEB, 2012	−30.7	1.4	−0.4	1.7
		Mean	0.6	2.0
		SD	0.8	1.3

## Data Availability

All datasets can be found within the Supplementary Material.

## Supplementary Materials

The following supporting information can be downloaded at: https://www.mdpi.com/article/10.3390/biology15030274/s1, Table S1a: Stable carbon and nitrogen isotope values of bamboos.; Table S1b: Stable carbon and nitrogen isotope values of giant panda feces; Table S2: Weighted diet of the giant panda during sampling period; Table S3a: Results of one-way ANOVA (Month) for δ^13^C, δ^15^N, C(%), N(%), and C:N in diet; Table S3b: Results of Tukey HSD post hoc pairwise comparisons for dietary δ^13^C, δ^15^N, C(%), N(%), and C:N between shoots and leaves of Bf and Fq; Table S3c: Results of independent samples *t*-test for δ^13^C, δ^15^N, C(%), N(%), and C:N between bamboo shoots and leaves; Table S4a: Results of one-way ANOVA (Month) for δ^13^C, δ^15^N, C(%), N(%), and C:N in feces; Table S4b: Results of Tukey HSD post hoc pairwise comparisons for feces δ^13^C, δ^15^N, C(%), N(%), and C:N between shoots and leaves of Bf and Fq; Table S4c: Results of independent samples *t*-test for δ^13^C, δ^15^N, C(%), N(%), and C:N in feces between bamboo shoots and leaves; Table S5: Results of Levene’s Test comparing isotopic (δ^13^C, δ^15^N) and elemental (C%, N%, C:N) composition between diet and feces; Figure S1: Monthly Changes in C:N Ratio of Bamboo Consumed by Giant Pandas from March 2011 to February 2012; Figure S2: The monthly diet–feces discrimination factors of the giant panda.

## Abbreviations

The following abbreviations are used in this manuscript:

SI	Stable isotope
SIA	Stable isotope analysis
DFs (Δ)	Discrimination factors
Bf	*Bashania fargesii*
Fq	*Fargesia qinlingensis*
