# Supplementary material for: Carbon and Nitrogen Stable Isotopic Discrimination Factors Between Diet and Feces in Wild Giant Pandas
Source: Biology (Basel). 2026 Feb 3;15(3):274. doi: 10.3390/biology15030274 (PMC12896708; doi:10.3390/biology15030274)
Supplement: Supplementary file 1 [file biology-15-00274-s001.zip › biology-4092025-supplementary.pdf]

Table S1a. Stable carbon and nitrogen isotope values of bamboos.

Month	n	δ13C diet (‰)	SD	δ15N diet (‰)	SD	C% diet	SD	N% diet	SD	C:N diet	SD
MAR,2011	8	-30.6	0.4	-1.2	1.2	47.6	0.9	2.6	0.3	18.5	1.1
APR,2011	14	-30.4	1.0	-1.7	0.7	47.1	1.7	2.6	0.3	18.1	1.2
MAY,2011	16	-24.4	0.6	-1.2	1.1	41.9	1.6	5.4	0.8	7.9	1.9
JUN,2011	6	-25.0	0.9	-0.7	1.0	43.5	1.4	3.9	0.6	11.1	2.1
JUL,2011	6	-26.1	0.3	0.8	0.5	43.9	2.1	3.2	0.8	13.8	1.1
AUG,2011	2	-30.5		-2.4		47.2		2.7		17.2	
SEP,2011	4	-29.6	1.1	-0.2	0.7	45.1	3.3	2.5	0.4	18.4	6.1
OCT,2011	10	-30.5	1.0	-0.2	1.3	47.8	1.6	2.8	0.2	17.0	0.6
NOV,2011	9	-30.3	0.8	-0.1	1.3	47.4	1.4	2.6	0.2	18.0	1.7
DEC,2011	2	-30.4		-0.8		48.2		2.6		18.6	
JAN,2012	3	-31.3	1.2	0.8	0.8	47.4	1.0	2.8	0.3	16.9	1.4
FEB,2012	3	-30.3	0.5	-0.3	1.2	47.2	1.2	2.5	1.2	18.6	2.3
Mean	83	-29.1	2.4	-0.6	0.9	46.2	2.1	3.0	0.8	16.2	3.5

Table S1b. Stable carbon and nitrogen isotope values of giant panda feces.

Month	n	δ13C feces (‰)	SD	δ15N feces (‰)	SD	C% feces	SD	N% feces	SD
MAR,2011	16	-30.2	1.1	0.5	1.6	44.4	1.6	1.6	0.4
APR,2011	14	-29.8	1.0	0.3	1.3	44.2	2.4	1.5	0.5
MAY,2011	20	-24.1	0.8	1.7	1.2	41.8	1.5	1.2	0.2
JUN,2011	8	-25.2	0.5	1.9	1.3	41.2	3.1	1.2	0.2
JUL,2011	8	-25.5	0.6	2.2	3.2	46.1	1.2	1.0	0.1
AUG,2011	2	-27.9		3.0		47.8		1.7	
SEP,2011	5	-29.1	0.7	1.3	0.7	46.3	1.0	2.0	0.2
OCT,2011	13	-29.8	0.8	1.6	1.1	46.2	1.9	2.0	0.3
NOV,2011	12	-29.7	0.8	1.3	1.6	45.8	1.1	1.9	0.3
DEC,2011	9	-30.2	0.8	1.7	1.1	44.9	1.8	1.9	0.3
JAN,2012	7	-30.3	0.4	1.3	1.5	44.8	0.9	1.9	0.2
FEB,2012	8	-30.7	0.8	1.4	1.7	44.0	1.3	1.6	0.1
Mean	122	-28.5	2.3	1.5	0.7	44.8	1.9	1.6	0.3

Table S2. Weighted diet of the giant panda during sampling period.

Food items	N	Portion of diet	δ^{13}C (‰)	SD	δ^{15}N (‰)	SD	C%	SD	N%	SD	C/N	SD
Bf leaves	53	66.70%	-30.4	0.9	-0.6	1.2	47.2	1.7	2.6	0.3	18.2	1.7
Bf shoots	22	16.70%	-24.6	0.7	-1.1	1.1	42.4	1.7	5.0	0.9	8.8	1.9
Fq shoots	6	8.30%	-26.1	0.3	0.8	0.4	43.9	2.0	3.2	0.7	14.9	5.9
Fq leaves	2	8.30%	-30.5		-2.4		47.2	1.1	2.7	0.0	17.2	0.3
**Weighted diet**	83		-29.1	0.6	-0.7	0.8	46.1	1.2	3.1	0.3	15.1	1.3

Table S3a. Results of one-way ANOVA (Month) for δ^{13}C, δ^{15}N, C (%), N (%), and C:N in diet.

Groups	Variable	df1	df2	F	p_value	Sig.
Month	δ13C diet (‰)	1	81	81.942	0.000	***
	δ15N diet (‰)	1	81	4.379	0.000	***
	C% diet	1	81	14.675	0.000	***
	N% diet	1	81	38.571	0.000	***
	C:N diet	1	81	27.192	0.000	***

Table S3b. Results of Tukey HSD post-hoc pairwise comparisons for dietary δ^{13}C, δ^{15}N, C (%), N (%), and C:N between shoots and leaves of Bf and Fq.

Variable	F	Comparison group	p_value	Sig.
δ^{13}C diet (‰)	739.397	Bf leaves & Bf shoots	0.000	***
	255.130	Fq leaves & Fq shoots	0.000	***
	0.035	Bf leaves & Fq leaves	0.852	ns
	25.016	Bf shoots & Fq shoots	0.000	***
δ^{15}N diet (‰)	1.973	Bf leaves & Bf shoots	0.164	ns
	27.593	Fq leaves & Fq shoots	0.002	**
	3.765	Bf leaves & Fq leaves	0.058	ns
	16.459	Bf shoots & Fq shoots	0.000	***
C% diet	123.349	Bf leaves & Bf shoots	0.000	***
	3.532	Fq leaves & Fq shoots	0.109	ns
	0.000	Bf leaves & Fq leaves	0.985	ns
	3.741	Bf shoots & Fq shoots	0.064	ns
N% diet	267.320	Bf leaves & Bf shoots	0.000	***

	0.572	Fq leaves & Fq shoots	0.478	ns
	0.466	Bf leaves & Fq leaves	0.498	ns
	17.434	Bf shoots & Fq shoots	0.000	***
C: N diet	429.221	Bf leaves & Bf shoots	0.000	***
	0.246	Fq leaves & Fq shoots	0.638	ns
	0.653	Bf leaves & Fq leaves	0.423	ns
	17.328	Bf shoots & Fq shoots	0.000	***

Table S3c. Results of independent samples t-test for $\delta^{13}C$, $\delta^{15}N$, C (%), N (%), and C:N between bamboo shoots and leaves.

Variable	t	df	p_value	Sig.
$\delta^{13}C$ diet (‰)	-26.374	52.6	0.000	***
$\delta^{15}N$ diet (‰)	-0.128	55.2	0.898	ns
C% diet	10.718	51.4	0.000	***
N% diet	-8.700	28.5	0.000	***
C:N diet	10.056	31.9	0.000	***

Table S4a. Results of one-way ANOVA (Month) for $\delta^{13}C$, $\delta^{15}N$, C(%), N(%), and C:N in feces.

Comparison Groups	Variable	df1	df2	F	p_value	Sig.
Month	$\delta^{13}C$ feces (‰)	11	110	97.446	0.000	***
	$\delta^{15}N$ feces (‰)	11	110	1.686	0.086	ns
	C% feces	11	110	10.748	0.000	***
	N% feces	11	110	15.738	0.000	***
	C:N feces	11	110	7.584	0.000	***

Table S4b. Results of Tukey HSD post-hoc pairwise comparisons for feces $\delta^{13}C$, $\delta^{15}N$, C (%), N (%), and C:N between shoots and leaves of Bf and Fq.

Variable	F	Comparison group	p_value	Sig.
$\delta^{13}C$ feces (‰)	781.506	Bf shoots & Fq shoots	0.000	***
	28.631	Fq leaves & Fq shoots	0.001	***
	10.390	Bf leaves & Bf shoots	0.002	**
	12.143	Bf leaves & Fq leaves	0.001	**
$\delta^{15}N$ feces (‰)	5.095	Bf shoots & Fq shoots	0.026	*

	0.092	Fq leaves & Fq shoots	0.770	ns
	3.409	Bf leaves & Bf shoots	0.068	ns
	0.444	Bf leaves & Fq leaves	0.510	ns
C% feces	68.733	Bf shoots & Fq shoots	0.000	***
	3.290	Fq leaves & Fq shoots	0.107	ns
	4.633	Bf leaves & Bf shoots	0.034	*
	35.792	Bf leaves & Fq leaves	0.000	***
N% feces	64.517	Bf shoots & Fq shoots	0.000	***
	37.626	Fq leaves & Fq shoots	0.000	***
	0.126	Bf leaves & Bf shoots	0.724	ns
	8.937	Bf leaves & Fq leaves	0.005	**
C:N feces	25.176	Bf shoots & Fq shoots	0.000	***
	9.420	Fq leaves & Fq shoots	0.015	*
	0.170	Bf leaves & Bf shoots	0.681	ns
	25.027	Bf leaves & Fq leaves	0.000	***

Table S4c. Results of independent samples t-test for $\delta^{13}C$, $\delta^{15}N$, C (%), N (%), and C:N in feces between bamboo shoots and leaves.

Variable	t	df	p_value	Sig.
$\delta^{13}C$ diet (‰)	-27.857	67.2	0.000	***
$\delta^{15}N$ diet (‰)	-2.207	54.9	0.032	*
C% diet	5.045	49.8	0.000	***
N% diet	11.932	105.3	0.000	***
C:N diet	-7.106	71.4	0.000	***

Table S5. Results of Levene's Test comparing isotopic ($\delta^{13}C$, $\delta^{15}N$) and elemental (C%, N%, C:N) composition between diet and feces.

Variable	Levene_F	Levene_p	Variance_Equal	t	df	p_value	Sig.
$\delta^{13}C$	0.466	0.495	Yes	-0.5	203	0.591	ns
$\delta^{15}N$	3.878	0.050	Yes	-9.9	203	0.000	***
C%	3.126	0.079	Yes	3.8	203	0.000	***
N%	25.061	0.000	No	12.4	95.21	0.000	***
C:N	10.082	0.002	No	-13.8	187.73	0.000	***

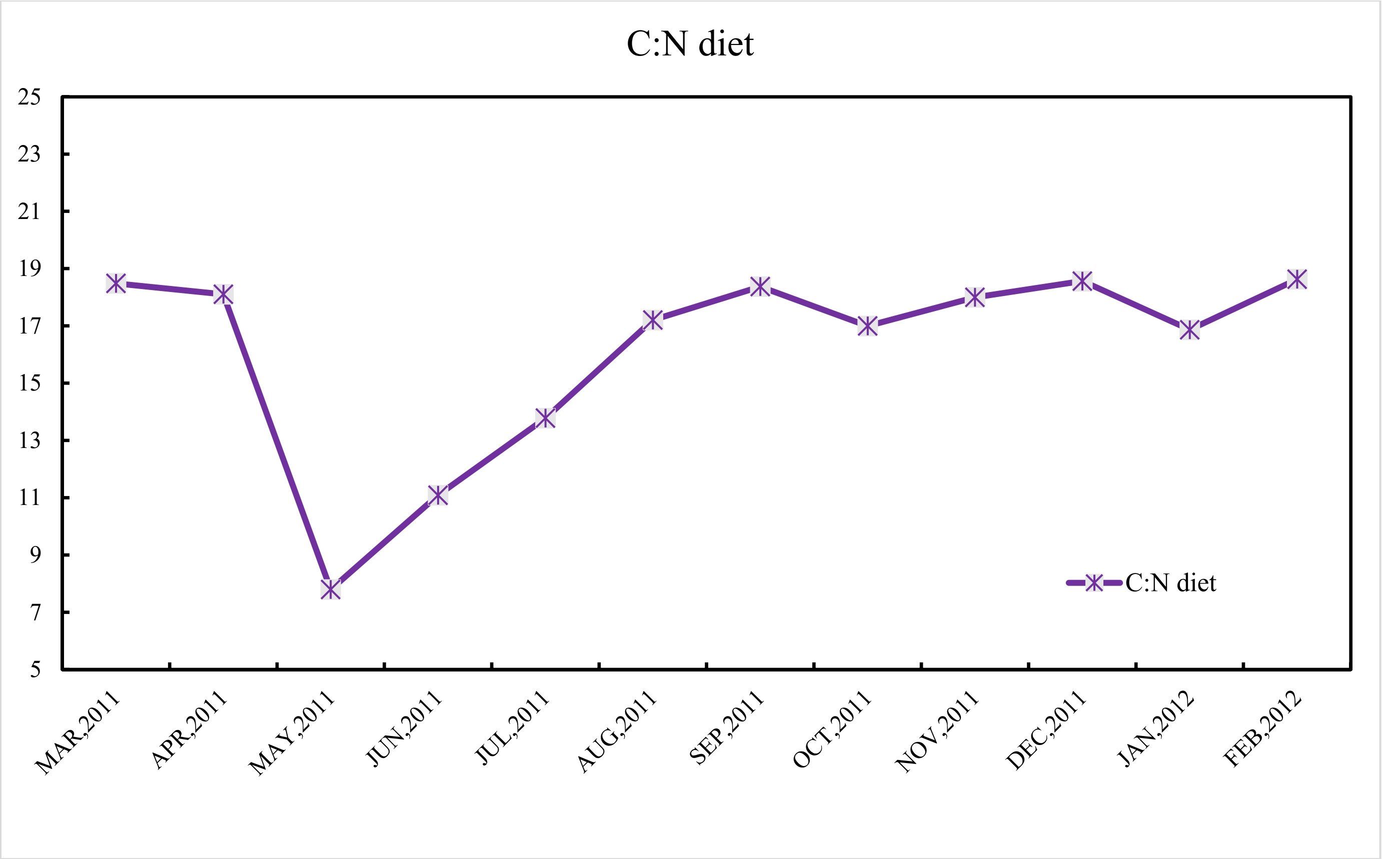

Figure S1. Monthly changes in C:N ratio of bamboo consumed by giant pandas from March 2011 to February 2012.

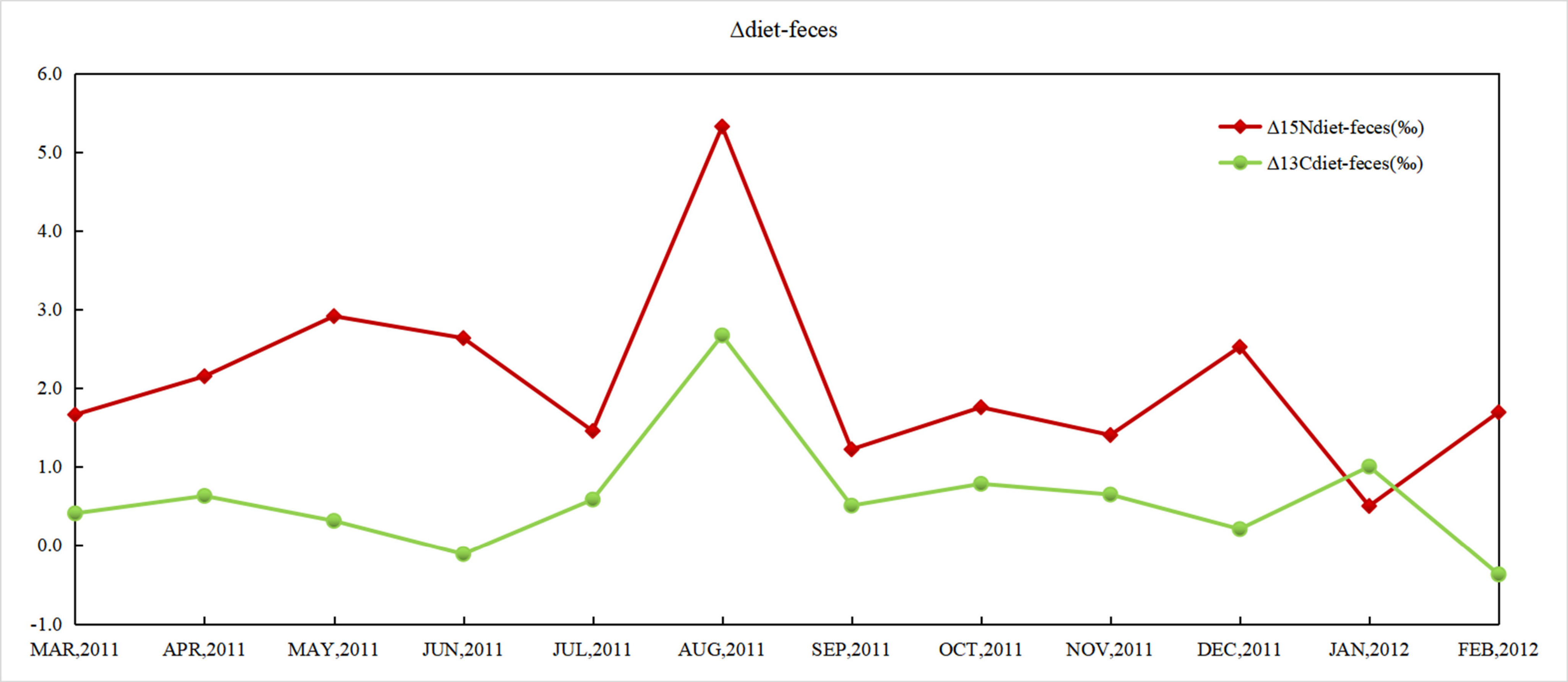

Figure S2 The monthly carbon and nitrogen diet-feces discrimination factors of the giant panda.
